# Bis(8-hy­droxy-1-methyl­quinolin-1-ium) bis­(1,2-dicyano­ethene-1,2-dithiol­ato)nickelate(II) dihydrate

**DOI:** 10.1107/S1600536811045430

**Published:** 2011-11-05

**Authors:** Zhi-Heng Guan, Zhang Jiang, Fang-Ming Wang

**Affiliations:** a School of Materials Science & Engineering, Jiangsu University of Science & Technology, Zhenjiang 212003, People’s Republic of China; b School of Biology & Chemical Engineering, Jiangsu University of Science & Technology, Zhenjiang 212003, People’s Republic of China

## Abstract

In the title ion-pair complex, (C_10_H_10_NO)_2_[Ni(C_4_N_2_S_2_)_2_]·2H_2_O, the anion has crystallographically imposed centre of symmetry. The Ni^II^ atom exhibits a slightly distorted square-planar coordination geometry. In the crystal, the water mol­ecule links anions and cations into a three-dimensional network *via* O—H⋯N, O—H⋯S and O—H⋯O hydrogen bonds. The structure is further stabilized by weak S⋯π contacts [S⋯centroid = 3.8047 (9) Å] and π–π stacking inter­actions [centriod–centroid distance = 3.8653 (7) Å].

## Related literature

For background to the properties and applications of bis-1,2-dithiol­ene metal complexes, see: Brammer (2004 ▶); Hill *et al.* (2005 ▶); Robin & Fromm (2006 ▶); Carlucci *et al.* (2003 ▶). For details of square-planar 1,2-dithiol­ene metal complexes, see: Robertson & Cronin (2002 ▶); Coomber *et al.* (1996 ▶); Ni *et al.* (2005 ▶); Duan *et al.* (2010 ▶).
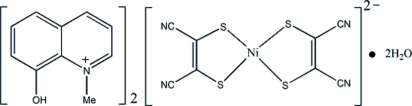


## Experimental

### 

#### Crystal data

(C_10_H_10_NO)_2_[Ni(C_4_N_2_S_2_)_2_]·2H_2_O
*M* *_r_* = 695.50
Triclinic, 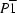

*a* = 8.786 (2) Å
*b* = 9.277 (2) Å
*c* = 9.667 (2) Å
α = 82.064 (4)°
β = 78.058 (4)°
γ = 83.324 (4)°
*V* = 760.4 (3) Å^3^
*Z* = 1
Mo *K*α radiation
μ = 0.96 mm^−1^
*T* = 291 K
0.35 × 0.20 × 0.15 mm

#### Data collection

Bruker SMART APEX CCD area-detector diffractometer
Absorption correction: multi-scan (*SADABS*; Bruker, 2000 ▶) *T*_min_ = 0.796, *T*_max_ = 0.865
3798 measured reflections
2679 independent reflections
2179 reflections with *I* > 2σ(*I*)
*R*_int_ = 0.057

#### Refinement

*R*[*F* ^2^ > 2σ(*F* ^2^)] = 0.041
*wR*(*F* ^2^) = 0.097
*S* = 1.00
2679 reflections
197 parameters
H-atom parameters constrained
Δρ_max_ = 0.52 e Å^−3^
Δρ_min_ = −0.28 e Å^−3^

### 

Data collection: *SMART* (Bruker, 2000 ▶); cell refinement: *SAINT* (Bruker, 2000 ▶); data reduction: *SAINT*; program(s) used to solve structure: *SHELXTL* (Sheldrick, 2008 ▶); program(s) used to refine structure: *SHELXTL*; molecular graphics: *SHELXTL*; software used to prepare material for publication: *SHELXTL*.

## Supplementary Material

Crystal structure: contains datablock(s) I, global. DOI: 10.1107/S1600536811045430/rz2659sup1.cif

Structure factors: contains datablock(s) I. DOI: 10.1107/S1600536811045430/rz2659Isup2.hkl

Additional supplementary materials:  crystallographic information; 3D view; checkCIF report

## Figures and Tables

**Table 1:** Hydrogen-bond geometry (Å, °)

*D*—H⋯*A*	*D*—H	H⋯*A*	*D*⋯*A*	*D*—H⋯*A*
O1—H1*W*⋯O2	0.98	1.66	2.639 (3)	180
O2—H2*B*⋯S2	0.85	2.64	3.224 (3)	128
O2—H2*B*⋯N2^i^	0.85	2.51	2.948 (3)	113

Symmetry code: (i) 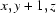.

**Fig. 1.**
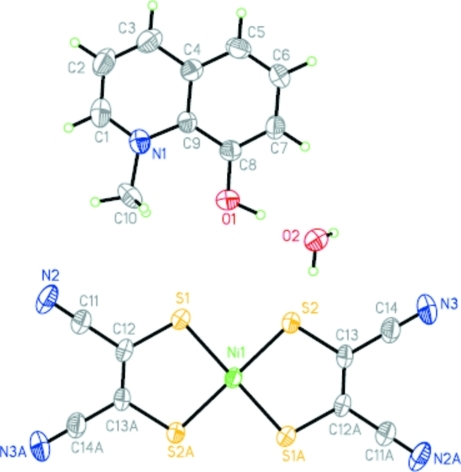
The molecular structure of the title compound, showing 30% probability displacement ellipsoids. Atoms labeled with the suffix A are generated by the symmetry operator (1 - x, -y, 2 - z).

## supplementary crystallographic information

### Comment

During the past few years, bis-1,2-dithiolene complexes of transition metals have been widely studied for their novel properties and applications in the areas of conducting and magnetic materials, dyes, non-linear optics, catalysis and others, due to their extended electronically delocalized core comprising the central metal, four sulphur atoms and the C═C units (Brammer, 2004; Hill *et al.*, 2005; Robin & Fromm, 2006; Carlucci *et al.*, 2003). Among them, maleonitriledithiolate (mnt^2-^) transition metal complexes are typical examples of bis-1,2-dithiolene complexes used as building blocks for magnetic and conducting molecular materials (Robertson & Cronin, 2002; Coomber *et al.*, 1996; Ni *et al.*, 2005; Duan *et al.*, 2010). To gain more insight into how the substituted groups affects the stacking mode of the Ni(mnt)_2_^2-^ anion, we herein present the crystal structure of a new Ni(mnt)_2_^2-^ salt containing the 1-methyl-8-hydroxyl-quinolinium (MeHoQl)^+^ cation.

As shown in Fig. 1,  the title salt consists of one (MeHoQl)^+^ cation, half of a Ni(mnt)_2_^2-^ anion and one water molecule in the asymmetric unit. The anion possesses crystallographically imposed inversion centre and exhibits a slightly distorted square-planar coordination geometry. The crystal structure is stabilized by O–H···N, O–H···S and O–H···O hydrogen bonds (Table 1) involving the water molecule, linking cations and anions into a three-dimensional network. Anions and cation further interact through weak S···π contacts (S2···Cg1^i^ = 3.8047 (9) Å) and π–π stacking interactions (Cg1···Cg2^ii^ = 3.8653 (7) Å; Cg1 and Cg2 are the centroids of the N1/C1–C4/C9 and C4–C9 rings, respectively; symmetry codes: (i) x, y, z-1; (ii) -x, -y, 1-z).

### Experimental

The title compound was prepared by the direct reaction of equimolar mixture of NiCl_2_.6H_2_O, sodium maleonitriledithiolate and 1-methyl-8-hydroxyl-quinolinium iodide in water/ethanol (1:1 *v*/*v*). Red-brown block-like single crystals were obtained by slow evaporation of a CH_3_CN solution at room temperature for about two weeks.

### Refinement

The hydroxy H atom was located in a difference Fourier map and refined as riding with O–H = 0.98 Å and free isotropic displacement parameter. All other H atoms were fixed geometrically and treated as riding with C—H = 0.96 Å, O–H = 0.85 Å and with *U*_iso_ (H) = 1.2*U*_eq_(C) or 1.5*U*_eq_(C, O) for methyl and water H atoms.

### Crystal data

**Table:**

(C_10_H_10_NO)_2_[Ni(C_4_N_2_S_2_)_2_]·2H_2_O	*Z* = 1
*M**_r_* = 695.50	*F*(000) = 358
Triclinic, *P*1	*D*_x_ = 1.519 Mg m^−^^3^
Hall symbol: -P 1	Mo *K*α radiation, λ = 0.71073 Å
*a* = 8.786 (2) Å	Cell parameters from 1684 reflections
*b* = 9.277 (2) Å	θ = 2.4–24.2°
*c* = 9.667 (2) Å	µ = 0.96 mm^−^^1^
α = 82.064 (4)°	*T* = 291 K
β = 78.058 (4)°	Block, red-brown
γ = 83.324 (4)°	0.35 × 0.20 × 0.15 mm
*V* = 760.4 (3) Å^3^	

### Data collection

**Table:**

Bruker SMART APEX CCD area-detector diffractometer	2679 independent reflections
Radiation source: sealed tube	2179 reflections with *I* > 2σ(*I*)
graphite	*R*_int_ = 0.057
φ and ω scans	θ_max_ = 25.0°, θ_min_ = 2.2°
Absorption correction: multi-scan (*SADABS*; Bruker, 2000)	*h* = −10→9
*T*_min_ = 0.796, *T*_max_ = 0.865	*k* = −11→10
3798 measured reflections	*l* = −11→8

### Refinement

**Table:**

Refinement on *F*^2^	Primary atom site location: structure-invariant direct methods
Least-squares matrix: full	Secondary atom site location: difference Fourier map
*R*[*F*^2^ > 2σ(*F*^2^)] = 0.041	Hydrogen site location: inferred from neighbouring sites
*wR*(*F*^2^) = 0.097	H-atom parameters constrained
*S* = 1.00	*w* = 1/[σ^2^(*F*_o_^2^) + (0.0419*P*)^2^] where *P* = (*F*_o_^2^ + 2*F*_c_^2^)/3
2679 reflections	(Δ/σ)_max_ < 0.001
197 parameters	Δρ_max_ = 0.52 e Å^−^^3^
0 restraints	Δρ_min_ = −0.28 e Å^−^^3^

### Special details

**Table:**

Geometry. All e.s.d.'s (except the e.s.d. in the dihedral angle between two l.s. planes) are estimated using the full covariance matrix. The cell e.s.d.'s are taken into account individually in the estimation of e.s.d.'s in distances, angles and torsion angles; correlations between e.s.d.'s in cell parameters are only used when they are defined by crystal symmetry. An approximate (isotropic) treatment of cell e.s.d.'s is used for estimating e.s.d.'s involving l.s. planes.
Refinement. Refinement of *F*^2^ against ALL reflections. The weighted *R*-factor *wR* and goodness of fit *S* are based on *F*^2^, conventional *R*-factors *R* are based on *F*, with *F* set to zero for negative *F*^2^. The threshold expression of *F*^2^ > σ(*F*^2^) is used only for calculating *R*-factors(gt) *etc*. and is not relevant to the choice of reflections for refinement. *R*-factors based on *F*^2^ are statistically about twice as large as those based on *F*, and *R*- factors based on ALL data will be even larger.

### Fractional atomic coordinates and isotropic or equivalent isotropic displacement parameters (Å^2^)

**Table:**

	*x*	*y*	*z*	*U*_iso_*/*U*_eq_	
C1	0.1931 (4)	−0.1818 (4)	0.2692 (4)	0.0795 (10)	
H1A	0.2548	−0.2742	0.2672	0.095*	
C2	0.0785 (4)	−0.1531 (4)	0.1891 (4)	0.0880 (12)	
H2A	0.0584	−0.2267	0.1362	0.106*	
C3	−0.0068 (4)	−0.0222 (4)	0.1882 (4)	0.0806 (10)	
H3A	−0.0890	−0.0007	0.1347	0.097*	
C4	0.0262 (3)	0.0846 (3)	0.2640 (3)	0.0593 (8)	
C5	−0.0561 (4)	0.2226 (4)	0.2571 (4)	0.0722 (9)	
H5A	−0.1334	0.2450	0.1983	0.087*	
C6	−0.0264 (4)	0.3248 (3)	0.3313 (4)	0.0724 (9)	
H6A	−0.0808	0.4207	0.3252	0.087*	
C7	0.0860 (3)	0.2922 (3)	0.4167 (3)	0.0648 (8)	
H7A	0.1040	0.3660	0.4704	0.078*	
C8	0.1707 (3)	0.1592 (3)	0.4260 (3)	0.0551 (7)	
C9	0.1429 (3)	0.0509 (3)	0.3479 (3)	0.0499 (7)	
C10	0.3498 (4)	−0.1336 (3)	0.4296 (4)	0.0816 (10)	
H10A	0.3917	−0.2316	0.4143	0.122*	
H10B	0.4315	−0.0692	0.3997	0.122*	
H10C	0.3070	−0.1299	0.5289	0.122*	
C11	0.5319 (4)	−0.3805 (3)	0.7603 (3)	0.0642 (8)	
C12	0.5454 (3)	−0.2796 (3)	0.8553 (3)	0.0529 (7)	
C13	0.3540 (3)	0.3089 (3)	1.0562 (3)	0.0533 (7)	
C14	0.2600 (4)	0.4443 (3)	1.0453 (4)	0.0684 (9)	
N1	0.2236 (3)	−0.0862 (3)	0.3466 (3)	0.0615 (7)	
N2	0.5227 (4)	−0.4590 (3)	0.6826 (3)	0.0914 (10)	
N3	0.1864 (4)	0.5531 (3)	1.0351 (4)	0.1009 (11)	
Ni1	0.5000	0.0000	1.0000	0.04543 (18)	
O1	0.2796 (2)	0.1319 (2)	0.5071 (2)	0.0766 (6)	
H1W	0.2823	0.2143	0.5595	0.108 (13)*	
O2	0.2867 (2)	0.3545 (2)	0.6480 (2)	0.0839 (7)	
H2B	0.3566	0.3340	0.6984	0.126*	
H2C	0.3081	0.4295	0.5889	0.126*	
S1	0.42937 (9)	−0.11519 (8)	0.84759 (9)	0.0614 (2)	
S2	0.33291 (10)	0.17888 (8)	0.95058 (9)	0.0642 (2)	

### Atomic displacement parameters (Å^2^)

**Table:**

	*U*^11^	*U*^22^	*U*^33^	*U*^12^	*U*^13^	*U*^23^
C1	0.082 (2)	0.059 (2)	0.094 (3)	−0.0075 (17)	0.002 (2)	−0.0232 (19)
C2	0.091 (3)	0.085 (3)	0.096 (3)	−0.014 (2)	−0.013 (2)	−0.044 (2)
C3	0.074 (2)	0.097 (3)	0.079 (3)	−0.014 (2)	−0.0163 (18)	−0.030 (2)
C4	0.0545 (17)	0.068 (2)	0.0558 (18)	−0.0078 (14)	−0.0072 (14)	−0.0133 (15)
C5	0.064 (2)	0.079 (2)	0.076 (2)	0.0047 (17)	−0.0257 (17)	−0.0060 (18)
C6	0.070 (2)	0.0572 (19)	0.091 (3)	0.0102 (15)	−0.0244 (19)	−0.0137 (18)
C7	0.0657 (19)	0.0527 (18)	0.078 (2)	0.0002 (15)	−0.0132 (17)	−0.0211 (16)
C8	0.0519 (17)	0.0595 (18)	0.0540 (18)	−0.0054 (14)	−0.0105 (14)	−0.0065 (14)
C9	0.0463 (15)	0.0481 (16)	0.0521 (17)	−0.0034 (12)	−0.0023 (13)	−0.0064 (13)
C10	0.070 (2)	0.066 (2)	0.102 (3)	0.0150 (16)	−0.0217 (19)	0.0055 (19)
C11	0.077 (2)	0.0518 (17)	0.068 (2)	−0.0134 (15)	−0.0130 (16)	−0.0142 (16)
C12	0.0634 (17)	0.0405 (15)	0.0569 (18)	−0.0102 (12)	−0.0069 (14)	−0.0161 (13)
C13	0.0656 (18)	0.0344 (14)	0.0603 (18)	−0.0057 (12)	−0.0091 (15)	−0.0109 (13)
C14	0.088 (2)	0.0467 (18)	0.073 (2)	−0.0078 (16)	−0.0168 (18)	−0.0117 (16)
N1	0.0581 (15)	0.0531 (15)	0.0690 (17)	−0.0041 (12)	−0.0024 (13)	−0.0080 (13)
N2	0.127 (3)	0.0699 (19)	0.091 (2)	−0.0243 (17)	−0.0265 (18)	−0.0361 (17)
N3	0.125 (3)	0.0481 (17)	0.131 (3)	0.0179 (17)	−0.039 (2)	−0.0167 (18)
Ni1	0.0547 (3)	0.0370 (3)	0.0474 (3)	−0.0059 (2)	−0.0121 (2)	−0.0100 (2)
O1	0.0821 (15)	0.0746 (15)	0.0849 (16)	0.0071 (11)	−0.0431 (13)	−0.0210 (13)
O2	0.0993 (17)	0.0704 (14)	0.0964 (18)	−0.0024 (12)	−0.0490 (14)	−0.0184 (13)
S1	0.0712 (5)	0.0514 (4)	0.0716 (5)	−0.0004 (3)	−0.0299 (4)	−0.0223 (4)
S2	0.0840 (6)	0.0460 (4)	0.0722 (5)	0.0070 (4)	−0.0361 (4)	−0.0195 (4)

### Geometric parameters (Å, °)

**Table:**

C1—N1	1.319 (4)	C10—H10A	0.9599
C1—C2	1.374 (5)	C10—H10B	0.9599
C1—H1A	0.9600	C10—H10C	0.9600
C2—C3	1.351 (5)	C11—N2	1.135 (3)
C2—H2A	0.9599	C11—C12	1.430 (4)
C3—C4	1.399 (4)	C12—C13^i^	1.334 (4)
C3—H3A	0.9600	C12—S1	1.735 (3)
C4—C5	1.396 (4)	C13—C12^i^	1.334 (4)
C4—C9	1.416 (4)	C13—C14	1.424 (4)
C5—C6	1.343 (4)	C13—S2	1.733 (3)
C5—H5A	0.9600	C14—N3	1.138 (4)
C6—C7	1.395 (4)	Ni1—S2^i^	2.1546 (8)
C6—H6A	0.9600	Ni1—S2	2.1546 (8)
C7—C8	1.366 (4)	Ni1—S1	2.1599 (7)
C7—H7A	0.9600	Ni1—S1^i^	2.1599 (7)
C8—O1	1.340 (3)	O1—H1W	0.9789
C8—C9	1.408 (4)	O2—H2B	0.8500
C9—N1	1.383 (4)	O2—H2C	0.8500
C10—N1	1.492 (4)		
			
N1—C1—C2	122.5 (3)	N1—C10—H10A	110.0
N1—C1—H1A	118.7	N1—C10—H10B	109.5
C2—C1—H1A	118.8	H10A—C10—H10B	109.5
C3—C2—C1	119.4 (3)	N1—C10—H10C	108.9
C3—C2—H2A	120.7	H10A—C10—H10C	109.5
C1—C2—H2A	119.8	H10B—C10—H10C	109.5
C2—C3—C4	119.9 (3)	N2—C11—C12	178.7 (4)
C2—C3—H3A	120.7	C13^i^—C12—C11	121.4 (3)
C4—C3—H3A	119.4	C13^i^—C12—S1	121.27 (19)
C5—C4—C3	120.0 (3)	C11—C12—S1	117.3 (2)
C5—C4—C9	120.6 (3)	C12^i^—C13—C14	122.4 (2)
C3—C4—C9	119.4 (3)	C12^i^—C13—S2	119.9 (2)
C6—C5—C4	120.2 (3)	C14—C13—S2	117.7 (2)
C6—C5—H5A	119.9	N3—C14—C13	179.1 (4)
C4—C5—H5A	119.9	C1—N1—C9	121.0 (3)
C5—C6—C7	119.8 (3)	C1—N1—C10	116.5 (3)
C5—C6—H6A	120.6	C9—N1—C10	122.6 (3)
C7—C6—H6A	119.6	S2^i^—Ni1—S2	180.00 (4)
C8—C7—C6	122.2 (3)	S2^i^—Ni1—S1	91.80 (3)
C8—C7—H7A	119.3	S2—Ni1—S1	88.20 (3)
C6—C7—H7A	118.6	S2^i^—Ni1—S1^i^	88.20 (3)
O1—C8—C7	120.8 (3)	S2—Ni1—S1^i^	91.80 (3)
O1—C8—C9	120.2 (3)	S1—Ni1—S1^i^	180.000 (1)
C7—C8—C9	119.0 (3)	C8—O1—H1W	111.5
N1—C9—C8	124.1 (3)	H2B—O2—H2C	109.5
N1—C9—C4	117.7 (3)	C12—S1—Ni1	103.10 (10)
C8—C9—C4	118.2 (3)	C13—S2—Ni1	103.92 (10)
			
N1—C1—C2—C3	0.2 (6)	C5—C4—C9—C8	1.4 (4)
C1—C2—C3—C4	2.5 (6)	C3—C4—C9—C8	−178.3 (2)
C2—C3—C4—C5	176.5 (3)	C2—C1—N1—C9	−1.6 (5)
C2—C3—C4—C9	−3.7 (5)	C2—C1—N1—C10	179.2 (3)
C3—C4—C5—C6	179.0 (3)	C8—C9—N1—C1	−179.0 (3)
C9—C4—C5—C6	−0.7 (5)	C4—C9—N1—C1	0.3 (4)
C4—C5—C6—C7	−0.7 (5)	C8—C9—N1—C10	0.2 (4)
C5—C6—C7—C8	1.3 (5)	C4—C9—N1—C10	179.5 (3)
C6—C7—C8—O1	178.9 (3)	C13^i^—C12—S1—Ni1	1.1 (3)
C6—C7—C8—C9	−0.6 (5)	C11—C12—S1—Ni1	−177.2 (2)
O1—C8—C9—N1	−0.9 (4)	S2^i^—Ni1—S1—C12	0.20 (10)
C7—C8—C9—N1	178.6 (3)	S2—Ni1—S1—C12	−179.80 (10)
O1—C8—C9—C4	179.8 (3)	C12^i^—C13—S2—Ni1	−2.0 (3)
C7—C8—C9—C4	−0.7 (4)	C14—C13—S2—Ni1	178.6 (2)
C5—C4—C9—N1	−178.0 (2)	S1—Ni1—S2—C13	−178.97 (10)
C3—C4—C9—N1	2.3 (4)	S1^i^—Ni1—S2—C13	1.03 (10)

Symmetry codes: (i) −*x*+1, −*y*, −*z*+2.

### Hydrogen-bond geometry (Å, °)

**Table:**

*D*—H···*A*	*D*—H	H···*A*	*D*···*A*	*D*—H···*A*
O1—H1W···O2	0.98	1.66	2.639 (3)	180.
O2—H2B···S2	0.85	2.64	3.224 (3)	128.
O2—H2B···N2^ii^	0.85	2.51	2.948 (3)	113.

Symmetry codes: (ii) *x*, *y*+1, *z*.
